# Polymorphonuclear Myeloid-Derived Suppressor Cells Are Abundant in Peripheral Blood of Cancer Patients and Suppress Natural Killer Cell Anti-Tumor Activity

**DOI:** 10.3389/fimmu.2021.803014

**Published:** 2022-01-18

**Authors:** Nicola Tumino, Francesca Besi, Stefania Martini, Anna Laura Di Pace, Enrico Munari, Linda Quatrini, Andrea Pelosi, Piera Filomena Fiore, Giulia Fiscon, Paola Paci, Francesca Scordamaglia, Maria Grazia Covesnon, Giuseppe Bogina, Maria Cristina Mingari, Lorenzo Moretta, Paola Vacca

**Affiliations:** ^1^ Immunology Research Area, Istituto di Ricovero e Cura a Carattere Scientifico (IRCCS) Bambino Gesù Children’s Hospital, Rome, Italy; ^2^ Unità Operativa (UO) Immunology, Istituto di Ricovero e Cura a Carattere Scientifico (IRCCS) Ospedale Policlinico San Martino, Genoa, Italy; ^3^ Pathology Unit, Istituto di Ricovero e Cura a Carattere Scientifico (IRCCS) Sacro Cuore Don Calabria, Negrar di Valpolicella, Italy; ^4^ Department of Molecular and Translational Medicine, University of Brescia, Brescia, Italy; ^5^ Institute for Systems Analysis and Computer Science “Antonio Ruberti”, National Research Council, Rome, Italy; ^6^ Department of Computer, Control and Management Engineering, Sapienza University of Rome, Rome, Italy; ^7^ Struttura Complessa (SC) Pneumologia Ospedale Villa Scassi, ASL3 Genovese, Genoa, Italy; ^8^ Experimental Medicine Department (DIMES), University of Genoa, Genoa, Italy

**Keywords:** natural killer, myeloid-derived suppressor cell, immunoscore, biomarker, lung tumor

## Abstract

Tumor microenvironment (TME) includes a wide variety of cell types and soluble factors capable of suppressing immune-responses. While the role of NK cells in TME has been analyzed, limited information is available on the presence and the effect of polymorphonuclear (PMN) myeloid-derived suppressor cells, (MDSC). Among the immunomodulatory cells present in TME, MDSC are potentially efficient in counteracting the anti-tumor activity of several effector cells. We show that PMN-MDSC are present in high numbers in the PB of patients with primary or metastatic lung tumor. Their frequency correlated with the overall survival of patients. In addition, it inversely correlated with low frequencies of NK cells both in the PB and in tumor lesions. Moreover, such NK cells displayed an impaired anti-tumor activity, even those isolated from PB. The compromised function of NK cells was consequent to their interaction with PMN-MDSC. Indeed, we show that the expression of major activating NK receptors, the NK cytolytic activity and the cytokine production were inhibited upon co-culture with PMN-MDSC through both cell-to-cell contact and soluble factors. In this context, we show that exosomes derived from PMN-MDSC are responsible of a significant immunosuppressive effect on NK cell-mediated anti-tumor activity. Our data may provide a novel useful tool to implement the tumor immunoscore. Indeed, the detection of PMN-MDSC in the PB may be of prognostic value, providing clues on the presence and extension of both adult and pediatric tumors and information on the efficacy not only of immune response but also of immunotherapy and, possibly, on the clinical outcome.

## Introduction

Lung tumor is the second most common cancer in both men and women (1). Almost 60% of all lung cancers are metastatic at diagnosis and metastases occur in various tissues and organs. Traditional therapeutic options for lung cancer treatment are surgery, chemotherapy and radiotherapy. However, given the overall poor prognosis, strategies to improve the efficacy of these treatments are strictly needed, especially for tumors in advanced stages (2). Significant therapeutic progresses have been achieved over the years leading to an improved prognosis. Thus, the 5-year survival probability of metastatic disease is now significantly higher but far from being satisfactory (3). In recent years, immunotherapy with checkpoint inhibitors revealed as a particularly promising approach, however anti-tumor immunity is frequently hampered by tumor-mediated immunosuppression and immune evasion which strongly compromise the clinical efficacy (2, 4–6). The impact of the tumor microenvironment (TME) has recently been emphasized also in the context of resistance to treatment (7, 8). Various types of immunosuppressive cells are involved, including tumor associated macrophages (TAMs) (9), regulatory T cells (Treg) (10), myeloid derived suppressor cells (MDSC) (11), mesenchymal stromal cells (MSC) (12). Among these cells, increasing attention has been paid on the effect of MDSC on the treatment and on the prognosis of lung cancer (13, 14).

MDSC represent a heterogeneous population composed of both immature and mature activated myeloid cells capable of inhibiting both innate and adaptive immune responses. Thus, it has been shown that both human and murine MDSC are capable of interfering with T and NK cell proliferation and/or function (15). On the basis of surface markers expression, human MDSC can be divided in two major subsets, namely, monocytic MDSC (Mo-MDSC) and polymorphonuclear MDSC (PMN-MDSC). Thus, Mo-MDSC are CD45^+^Lin^-^(CD3^-^CD19^-^CD56^-^) HLA-DR^−/low^CD33^+^CD11b^+^CD14^+^CD15^−^CD66b^-^ while PMN-MDSC are CD45^+^Lin^-^HLA-DR^−/low^CD33^+^CD11b^+^CD14^-^CD15^+^CD66b^+^ (16).

Expansion/accumulation of these immunosuppressive cells may be due to a partial block in their differentiation from immature myeloid cells. An expansion of MDSC during acute/chronic viral or bacterial infection has recently been reported (17). In addition, previous studies revealed the presence, in the TME of different tumors, of suppressive cell types that compromise anti-tumor immune responses and favor the expansion of MDSC (18–26). Importantly, both Mo- and PMN-MDSC have been detected at the tumor site and even in peripheral blood (PB) of tumor patients (27–29). Notably, their presence has been associated with a poor prognosis (20). In addition, they have been detected in the PB of patients with sepsis or GvHD and also in healthy donors who received G-CSF for HSC mobilization (27).

MDSC have been shown to suppress immune cells by different mechanisms. For example, nitric oxide synthase 2 (NOS2) is produced by Mo-MDSC while reactive oxygen species (ROS) by PMN-MDSC. Together with arginase 1 (ARG1), they induce suppression of T cell proliferation consequent to inhibition of CD3ζ chain expression and to induction of T cell apoptosis. Additional mechanisms of MDSC-mediated immunosuppression are due to Indoleamine 2,3-dioxygenase (IDO)-derived L-kynurenine (a tryptophan catabolite), and to prostaglandin E2 (PGE2), which cause both T and NK cell dysfunction (19, 30). Also cytokines produced by MDSC, such as TGF-β and IL-10, have been shown to inhibit NK cell cytotoxicity and to induce Treg (31). As recently reported, another mechanism by which MDSC may exert suppression is by secreting exosomes, known as important players in intercellular communications (32, 33).

NK cells play a relevant role in the control of tumor growth and metastatic spread (34). They are able to efficiently kill tumor and virally-infected cells thanks to their ability to release cytolytic granules and pro-inflammatory cytokines (35). However, these NK-mediated effector functions can be compromised by cells or soluble factors present in TME. In this context, in a previous study we showed that PMN-MDSC derived from G-CSF-mobilized donors, undergoing apheresis for hematopoietic stem cell transplantation (HSCT), are able to strongly suppress the anti-tumor cytotoxicity and cytokine production of NK cells, thus compromising their important role in graft versus leukemia activity (GvL) (27, 36, 37).

In the present study, we show that the subset of PMN-MDSC is present not only at the tumor site, but also in the PB of patients with primary or metastatic lung tumors. Since these cells were enriched in cancer patients, they could represent a useful marker revealing tumor presence. Importantly, they impact on the frequency of mature NK cells present in patient’s PB and compromise their function. This inhibitory effect is primarily mediated, by cell-to-cell contact and PMN-MDSC-derived exosomes. It is conceivable that PMN-MDSC may play a primary role in the inhibition of the NK-mediated anti-tumor activity. Moreover, this effect may impair immunotherapy and the overall survival of patients with cancer as also suggested by our recent study in pediatric patients with neuroblastoma. This study offers an important clue for therapeutic interventions focused on targeting PMN-MDSC in order to block their immunosuppressive activity in tumor tissues and also in the periphery.

## Results

### Presence of PMN-MDSC in TME and PB of Patients With Primary or Metastatic Lung Tumor

**Figure 1:**
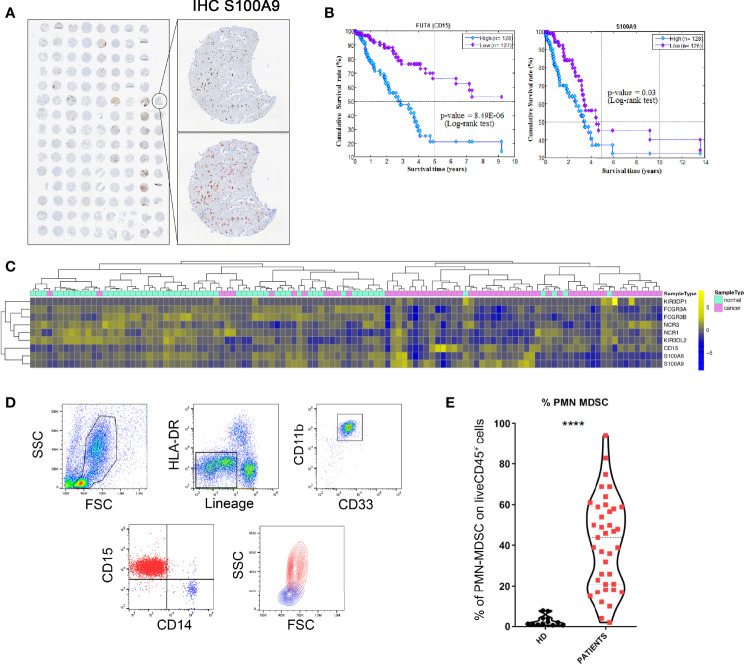
PMN-MDSC are present in high frequency in PB of patients with primary and metastatic lung tumor and correlate with their overall survival. **(A)** TMA of lung adenocarcinoma cases stained with S100A9 showing positive cells digitally quantified within tissue cores. The magnification shows S100A9 (upper panel) and S100A9^+^ cells automatically indicated with red circles (lower panel) to perform a cell count in each core using QuPath (absolute numbers of S100A9^+^ per mm^2^). **(B)** Kaplan-Meier survival analysis. Kaplan-Meier analyses to evaluate the correlations between the expression level of CD15 and S100A9 and the Overall Survival in lung adenocarcinoma patients retrieved from TCGA. Low- and high-expression groups refer to patients with expression levels lower than the 25^th^ percentile (violet curve) and greater than the 75^th^ percentile (cyan curve), respectively. **(C)** Hierarchical clustering and heatmap for KIR3DP1, FCGR3A, FCGR3B, NCR3, NCR1, KIR3DL2, CD15, S100A8, and S100A9 in 57 lung adenocarcinoma samples (violet bars) and 57-matched normal samples (water blue bars) retrieved from TCGA. The expression profiles of genes are clustered according to rows (genes) and columns (samples) by using the Pearson correlation as the distance metric and the complete-linkage as clustering method. The colors represent different expression levels that increase from blue to yellow. **(D)** PBMC were analyzed *ex-vivo* by flow cytometry for the expression of specific markers that allow the identification of PMN-MDSC. One representative experiment out of 34 performed. **(E)** Percentages of PMN-MDSC (CD15^+^ cells) in the PB of healthy donors (HD) and lung tumor patients (n=34). ****p ≤ 0.0.00005.

TME may contain different cell types capable of inhibiting the anti-tumor activity of effector cells, thus favoring immune evasion and tumor growth (38). In particular, we assessed whether MDSC were present in the TME of lung tumors using a tissue microarray (TMA). Twenty different tumor tissue samples from patients with lung adenocarcinoma, were analyzed by immunohistochemistry (IHC) for the expression of S100A9, a suitable marker for PMN-MDSC identification (39). As shown in **Figure 1A**, in the majority of these samples (14 out of 20) S100A9^+^ cells were highly represented (cell mean > 230 per mm^2^ of S100A9^+^ cells, from 70 to 909 per mm^2^ of S100A9^+^ cells) while in the other 6 cases were present in lower but sizable percentages (cell mean range from 21 to 55 per mm^2^ of S100A9^+^ cells). These results indicate that PMN-MDSC accumulate in TME where they may exert immunosuppressive activity on tumor-infiltrating immune effector cells.

The Overall Survival analysis performed by using the Kaplan Meier method on lung adenocarcinoma patients retrieved from TCGA revealed that a higher expression of S100A9 significantly correlates with a poor clinical outcome. Notably similar results were observed analyzing CD15 marker expression indicating a possible association between CD15^+^ or S100A9^+^ cells and poor prognosis (**Figure 1B**). These data suggest that CD15 and S100A9, markers both strictly related to PMN-MDSC cells, were involved in the overall survival of lung tumor patients. We also performed the expression analysis of markers (i.e. NCR1, FCGR3A, FCGR3B, NCR3, KIR3DL2, KIR3DP1), specific for NK cells using the same dataset. As shown in **Supplementary Figure 1A**, it is possible to speculate that a higher accumulation of NK cells occurred in normal as compared to lung tumor tissues. We also analyzed the expression of S100A8, S100A9 and CD15, myeloid markers that individually cannot specifically identify PMN-MDSC. S100A9 and CD15 are both partially down-regulated in normal samples with respect to lung tumor samples while S100A8 is higher in normal tissues (**Supplementary Figure 1A**). Moreover, the hierarchical clustering and heatmap for genes specific for NK cells and PMN-MDSC in 57 lung adenocarcinoma samples and 57-matched normal samples retrieved from TCGA indicated that genes specific for NK cells were enriched in normal tissue while genes specific (S100A9 and CD15) for PMN-MDSC were more expressed in the tumor tissues (**Figure 1C**).

In a second set of experiments, we investigated whether PMN-MDSC were present in PB of patients with primary or metastatic lung or pleural tumors. To this end, we applied a gating strategy allowing to identify and characterize, by flow-cytometry, the different MDSC subsets (16). In particular, we analyzed the PB of 34 patients with tumors (**Table 1**) for the presence of PMN-MDSC identified as CD45^+^ Lin^-^ HLA-DR^low/-^ CD11b^+^ CD33^+^ CD14^-^ CD15^+^ (**Figure 1D** and **Supplementary Figure 1B**). As shown in **Figure 1E**, we could detect relevant proportions of PMN-MDSC in the PB of tumor patients while these cells were virtually absent in PB of healthy donors (HD) indicating a possible correlation between the presence of PMN-MDSC in PB and the occurrence of a cancer.

**Table 1:** Features of patients included in the study.

***Patients with primary lung or pleural tumor***
*EpitheIioid mesothelioma*
Female: n. 3 - median age 76 (range 68-86)
Male: n. 6 - median age 72,5 (range 60-82)
*Non-small cell lung cancer*
Female: n. 7 - median age 69.4 (range 60-81)
Male: n. 6- median age 70.3 (range 58-77)
*Small cell lung cancer*
Male: n. 2 - median age 70.5 (range 69-72)
n- 24 - female: 10m male: 14 –median age 71.7 (range 58-86)
***Patients with secondary metastatic lung plural tumor***
*Intestinal Carcinoma*
Male: n. 1 - age 74
*Uterine Carcinoma*
Female: n. 1 - age 63
*Chorio-Carcinoma*
Female: n. 1 - age 41
*Vescical Carcinoma*
Female: n. 1 - age 57
*Brest Carcinoma*
Female: n. 2 - median age 57.5 (range 53-62)
*Kidney Carcinoma*
Male: n. 1 - age 73
*Primary tumor unknow*
Female: n. 2 - median age 83 (range 80-86)
Male: n. 1 - age 64
n. 10 -female: 7, male: 1- median age 65.3 (range 41-86)

### PB-Derived PMN-MDSC Inhibit the Anti-Tumor Activity of NK Cells

**Figure 2:**
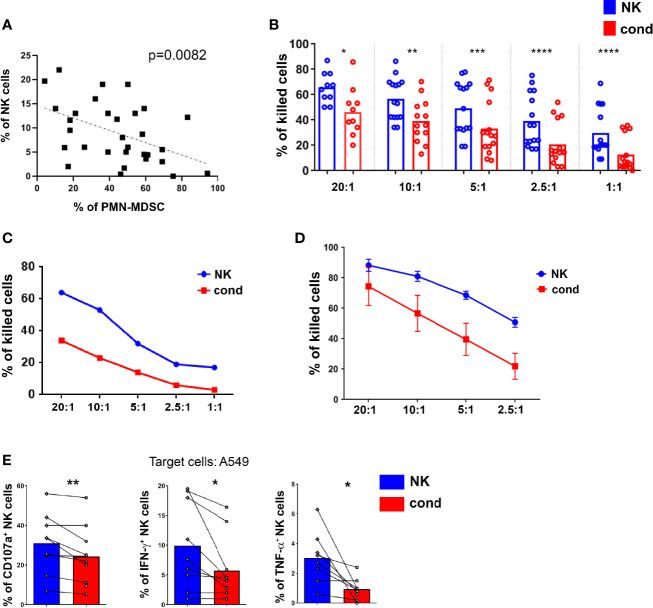
PMN-MDSC impair the anti-tumor activity of NK cells. **(A)** Correlation between the frequencies of PMN-MDSC and NK cells present in PB of lung tumor patients. P = 0.0082 (n = 31) **(B–E)** NK cells activated *in vitro-*expanded or *ex vivo* were cultured alone (NK) or in the presence (ratio 1:1) of PMN-MDSC (cond.) derived from PB of lung tumor patients. After 48h of co-culture, PMN-MDSC were depleted from 1:1 co-cultures and the resulting NK cells used as effector cells in the different functional assays. **(B, C)** Percentages of killed NALM-18 target cells. **(B)** Statistical analysis of 15 independent experiments. **(C)** One representative experiment out of 15 performed. **(D)** Percentages of killed NALM-18 target cells of short-term NK cells conditioned or not with PMN-MDSC derived from PB of lung tumor patients (n = 3). The different Effector/Target (E/T) ratios are indicated. **(E)** Cytokine production and degranulation capabilities of NK and cond. cells were analyzed after 4h of co-culture with A549 target cells. Bars indicated percentage of median of cytokines production (IFN-γ and TNF-α) and degranulation (CD107a) of NK and cond. cells (n = 9). *p ≤ 0.05; **p ≤ 0.005; ***p ≤ 0.0005; ****p ≤ 0.0.00005.

Since NK cells are known to display an important role in the anti-tumor activity, we first investigated a possible association between PB-PMN-MDSC and NK cells in our cohort of patients by correlating the proportion of PMN-MDSC and that of NK cells. As shown in **Figure 2A**, a statistically significant inverse correlation exists between the percentages of PMN-MDSC and that of NK cells. This result suggested that, in our cohort of tumor patients, PMN-MDSC could exert their immunosuppressive activity also by influencing the proportion of circulating NK cells.

We further analyzed whether PMN-MDSC could impair the anti-tumor activity of effector cells not only at the tumor site, but also in peripheral tissues. In order to analyze in more detail the immunosuppressive activity of PMN-MDSC, these cells were isolated from PB of tumor patients and co-cultured with fresh, short-term and long-term IL2-activated allogenic NK cells at 1:1 ratio, referred in the text as “conditioned” NK cells (cond.). After 48 hours, NK cells were isolated from co-cultures (by magnetic depletion of CD15^+^ cells) and assessed for their cytolytic activity against tumor cells. Notably, in order to better mimic the possible inhibitory effect of PMN-MDSC in the lung TME, primitive tumor cells, isolated from mesothelioma and adenocarcinoma patients, were also used. NK cells cultured in the absence of PMN-MDSC were comparatively analyzed. As shown in **Figures 2B, C** and **Supplementary Figure 1C**, target cell killing was strongly inhibited in conditioned NK cells analyzed at different effector:target (E:T) ratios. In addition, as shown in **Figure 2D**, PMN-MDSC could also inhibit the cytolytic activity of short-term-IL-2 activated allogenic PB-NK cells. Next, we investigated the effect of patient’s PMN-MDSC on autologous NK cells. To this end, both PMN-MDSC and NK cells were isolated from PB. These PMN-MDSC displayed a strong inhibitory effect on the cytotoxicity of autologous NK cells (**Supplementary Figure 1D**). We then assessed whether PMN-MDSC could inhibit also the production of pro-inflammatory cytokines and the degranulation (CD107a expression) of NK cells. Results indicate that a sharp reduction of IFN-γ, TNF-α and CD107a expression occurred also in conditioned NK cells (**Figure 2E** and **Supplementary Figure 1E**). Taken together, these results suggest that, in patients with lung tumor, PB-PMN-MDSC can compromise the anti-tumor effector function of NK cells.

### PMN-MDSC Influence the Gene Expression Profile of PB-NK Cells in Lung Tumor Patients

**Figure 3:**
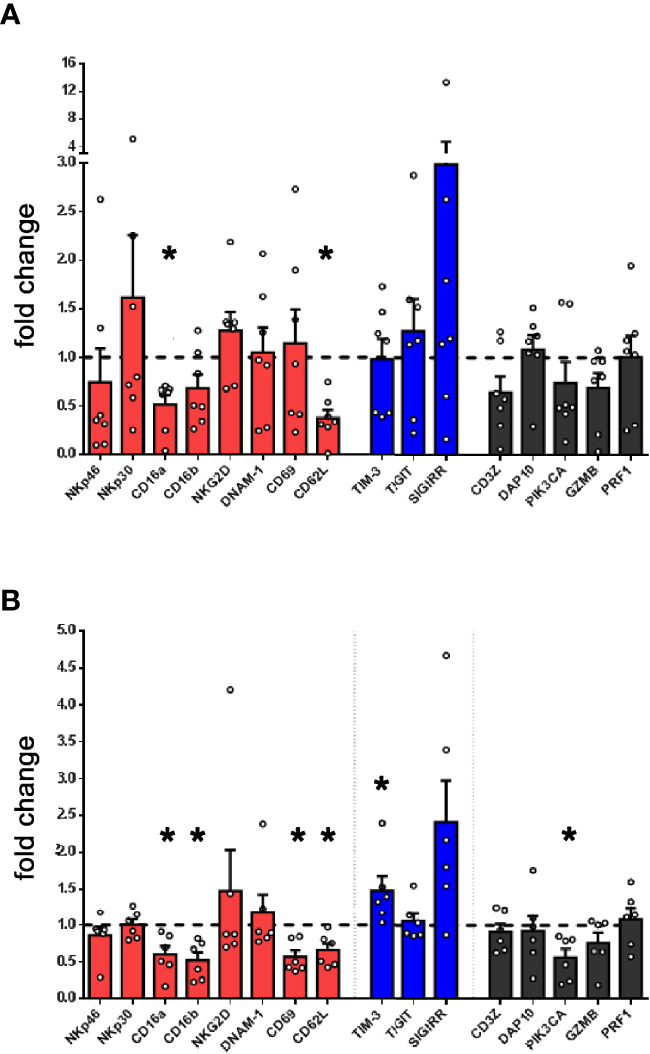
Lung tumor NK cells display an impairment of their activation status and inversely correlate with PMN-MDSC. **(A)** The expression of a panel of selected genes associated to NK cell function was evaluated by PCR array in PB-NK cells derived from lung cancer patients (n = 7). Values are expressed as fold change with respect to NK cells from HD (n = 5). Bars represent SEM. **(B)** The expression of genes associated to NK cell function was evaluated in short-term IL-2 activated NK cells upon co-culture with lung cancer PMN-MDSC. Values are expressed as fold change with respect to NK cells cultured with IL-2 only, used as control (n=6). *p ≤ 0.05.

Since PMN-MDSC, present in high numbers in PB of tumor patients, exert a potent inhibitory activity on NK cells, we investigated whether NK cells present in patient’s PB were modified in their gene expression profile. Thus, by PCR array, we compared the expression of a wide panel of genes involved in NK cell biology between PB-NK cells from patients and HD. In particular, we focused on genes involved in NK cell immuno-effector function or in their development. As shown in **Figure 3A**, patient-derived NK cells showed a markedly decreased expression of CD16 and a trend of decreased expression of several other genes known to play a role in NK-mediated anti-tumor activity, including genes encoding for NKp46, CD69, CD62L, DAP10 and GZMB. Of note, in patient-derived PB-NK cells, a higher expression of the inhibitory receptor TIGIT was observed.

To verify the contribution of PMN-MDSC on the peculiar transcriptional profile of patient’s NK cells, we further analyzed the gene expression of IL-2-activated allogenic HD-NK cells conditioned or not with patient-derived PMN-MDSC. Notably, conditioned NK cells displayed a decrease expression of several genes associated to NK cell activity as compared to unconditioned ones (**Figure 3B**). On the other hand, upon conditioning, the checkpoint inhibitor SIGIRR (40) resulted slightly increased. Similar data trend were observed by analyzing in flow cytometry the expression of proteins encoded by some of these genes (**Supplementary Figures 2A, B**). Despite some differences, a similar trend was observed in gene expression by NK cells isolated from patients confirming that PMN-MDSC present in PB of lung tumor patients may represent a cellular player responsible for TME-induced immunosuppression.

### Exosomes and Cell-to-Cell Contact Are Involved in the Inhibition of the NK-Mediated Anti-Tumor Cytolytic Activity of NK Cells

**Figure 4:**
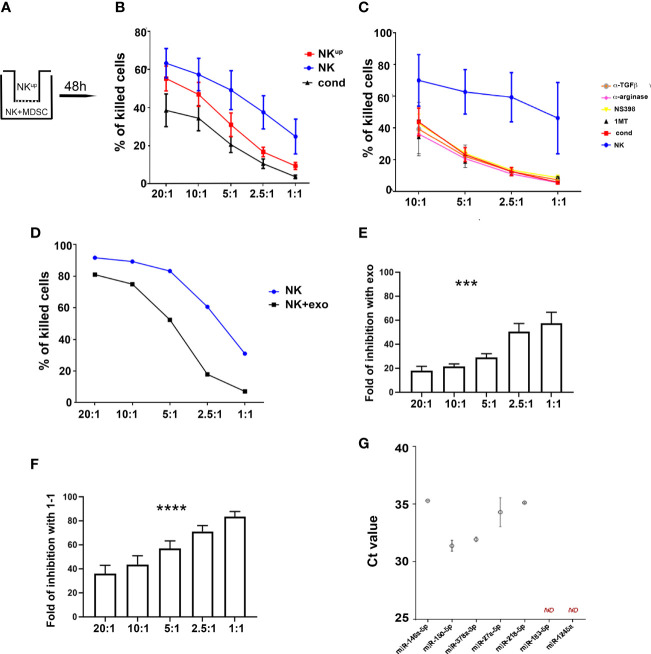
PMN-MDSC-mediated inhibitory mechanisms of NK cell function. **(A–C)** Activated NK cells were cultured alone (NK) or with PMN-MDSC (ratio 1/1) under cell-to-cell contact or transwell (NK^up^) condition. After 48h of co-culture, PMN-MDSC were depleted in the 1/1 condition and the resulting NK cells were used as effector cells (cond.) in the functional assays. **(A)** Schematic culture conditions. **(B)** Percentages (mean ± SEM) of killed NALM-18 target cells at different E/T ratios in NK cells cultured alone (blue), in cell-to-cell contact with PMN-MDSC (black) and in the transwell chamber (red) (n = 6). **(C)** Percentages of mean ± SEM of killed NALM-18 target cells by NK cells cultured alone or in the presence of PMN-MDSC either in the absence or in the presence of indicated inhibitors and blocking mAbs (n = 3). **(D)** Activated NK cells were incubated with 20 ug of exo-derived PMN-MDSC. After 48h their cytolytic potential was assessed against NALM-18 cell line. Percentages of mean ± SEM of killed NALM-18 target cells at different E/T ratios. One representative experiment. **(E)** Fold of inhibition of NK cell killing capability upon incubation with 20ug of PMN-MDSC derived exosomes at different E/T ratios (n = 4). **(F)** Fold of inhibition of NK cell killing capability upon co-culturing with PMN-MDSC at different E/T ratios (n = 9). **(G)** Expression of immuno-modulatory miRNAs in lung cancer PMN-MDSC-derived exosomes. Real time PCR analysis for the indicated miRNAs in lung cancer PMN-MDSC exosomes. Threshold cycle (Ct) values for each miRNA are reported. Bars indicate SD (n = 2). ND, Non Detected. ***p ≤ 0.0005; ****p ≤ 0.0.00005.

Previous studies showed that PMN-MDSC can suppress immune cell function by exploiting different mechanisms, including cell-to-cell contact and release of soluble factors (41). In order to identify the nature of the inhibitory mechanism(s) that impair NK-cell function, a first set of experiments was performed using trans-well chambers as illustrated in **Figure 4A**. The cytolytic activity was partially inhibited under trans-well conditions, suggesting that a soluble mechanism is involved in NK cell-mediated suppression (**Figure 4B**). Previous studies provided evidence that NK cell function may be compromised by IDO-derived catabolites (in particular L-kynurenine) and/or PGE2 (42) and TGF-β. Thus, co-culture experiments were performed using competitive inhibitors or blocking antibodies specific for IDO, PGE2, and TGF-β as well as for other inhibitory factors known to be released by PMN-MDSC (e.g. Arginase, catalase, nitric-oxide synthase). As shown in **Figure 4C**, none of these inhibitory pathways was involved in PMN-MDSC-mediated immunosuppression of NK cells. As shown in **Supplementary Figure 2C**, PMN-MDSC derived from lung tumor patients expressed different molecules that could be involved in modulation of immunoresponse (43–45). In addition, we further evaluated the involvement of other soluble elements and, in particular, we investigated the possible effect of exosomes released by PMN-MDSC (33). To this end, PMN-MDSC isolated from patient’s PB were cultured for 48 hours in exosome-free-media. Exosomes were then isolated from supernatants by ultracentrifugation. Flow cytometric analysis confirmed their expression of the exosome-specific markers CD81 and CD63 (data not shown). To assess their immunomodulatory potential, IL-2-activated HD-NK cells were cultured either in the presence or in the absence of PMN-MDSC derived-exosomes. The cytolytic activity of NK cells cultured in the presence of exosomes was significantly lower than that of NK cells cultured alone (**Figure 4D**). Notably, the degree of inhibition mediated by either exosomes (**Figure 4E**) or cell-to-cell contact (**Figure 4F**) was comparable at different E:T ratios. All these results suggested that PMN-MDSC-derived-exosomes represent important inhibitory mediators on anti-tumor NK cell function. In addition, we observed that PMN-MDSC-derived exosomes contain a set of miRNA (**Figure 4G**) with immunomodulatory properties (46).

## Discussion

NK cells have been shown to play an important role in the control of viral infections and tumor growth and metastases. Despite their strong anti-tumor activity, in tumor patients their effector function is frequently impaired by soluble inhibitory factors and/or immunosuppressive cells present in TME. Of note, evaluation of the immune infiltrate (immunoscore) in tumor lesions represents a valuable tool to stratify patients in different prognostic categories (47–49). However so far, immunomodulatory cells detectable in TME, have not been included in the immunoscore. MDSC represent a cell population derived from a common myeloid precursor present in the bone marrow. Under pathological conditions characterized by the presence of inflammation, a partial block of myeloid differentiation may favor the accumulation of MDSC, both in PB and tissues. Although different subsets of MDSC display morphological heterogeneity, they share the ability to suppress both innate and adaptive immune responses. MDSC exert a potent immunosuppressive activity and their presence has been documented in tumor patients (18–20, 50). Primary inflammatory cytokines such as IL6 and IL1β that may be present at high levels in TME and may drive the accumulation of MDSC favoring their immunosuppressive activity.

In this study, we show that PMN-MDSC are present not only in TME but also in PB of patients with primary or metastatic lung or pleural tumors. Remarkably, the detection and the numbers of PMN-MDSC in PB of these patients (since they are virtually absent in HD) may provide a clue for the presence and, possibly, the progression of a tumor. Of note PMN-MDSC could exert a strong inhibitory activity on NK cells in the periphery, further compromising the anti-tumor activity of these potent effector cells. In this context, we show that PMN-MDSC isolated from patients PB can exert a potent inhibitory effect *in vitro* on NK cell cytotoxicity, degranulation and cytokine production. Remarkably, the existence of an inverse correlation between the frequencies of PMN-MDSC and that of NK cells in the PB of lung tumor patients was compatible with the concept that the inhibitory effect may occur also in peripheral tissues. In addition, a significant correlation exists between the high frequency of PMN-MDSC and a poor clinical outcome in lung tumor patients.

Our data clearly show that even NK cells isolated from PB of tumor patients are impaired in their functional activities. Of note, they exhibit a peculiar gene expression profile. These data are in agreement with the downregulation of major activating NK receptors and consequent impaired anti-tumor effector function. Importantly, we provide evidence that these changes in gene expression profile can be induced by the interaction with PMN-MDSC.

Previous studies revealed different mechanisms by which PMN-MDSC suppress immune cells including release of soluble factors and cell-to-cell contact. Of note, we could not detect the occurrence of classical immunosuppressive mechanisms including Arginases, NO, IDO, TGF-β, PGE2 and ROS (51). Since exosomes were recently reported as an additional immunomodulatory mechanism, we assessed the possible contribution of PMN-MDSC-derived exosomes (51). Indeed, exosomes produced by PB-PMN-MDSC, isolated from lung tumor patients, contain a set of miRNA with immunomodulatory properties (46) that could inhibit the cytolytic activity of NK cells. These data indicate that the immune-modulatory activity of PMN-MDSC in lung tumor patients may be exerted both by their direct contact with effector cells and by the release of exosomes.

Of note, PMN-MDSC are present in the PB of pediatric patients affected by tumors with severe prognosis, such as neuroblastoma, and may compromise the effectiveness of immuno-therapies (i.e. chimeric antigen receptor-T cells, CAR-T cells, directed to GD2 antigen) (52). These findings further underscore the need to antagonize or targeting these cells to achieve a successful therapy.

In conclusion, our data clarified an important mechanism of immunosuppression occurring in primary and metastatic lung or pleural tumors, offering a clue to implement the immunoscore with the evaluation of PMN-MDSC numbers at the tumor lesion. The inhibitory effect of PMN-MDSC present in PB in normal versus pathological conditions reflects major differences in frequency rather than qualitative differences in the suppressive capacity of PMN-MDSC (**Supplementary Figure 2D**) and of their exosomes. Thus, identification of these cells in PB may provide a novel marker revealing the presence/extension of a tumor. Moreover, the assessment of their size in PB could indeed contribute to provide useful information on the clinical status and on prognostic aspects in different tumors (52). Importantly, it is also conceivable that targeting PMN-MDSC may offer a new strategy in the treatment of these type of tumors, complementary to other immune-therapies, including the use of checkpoint inhibitors or CAR-T, contributing to restore effective anti-tumor responses.

## Materials and Methods

### Samples, and Ethical Statements

34 patients with primary or metastatic lung tumor were enrolled at ASL3, Ospedale Villa Scassi, Genoa, Italy and analyzed at the time of diagnosis. Details on patient characteristics are summarized in **Table 1**. Peripheral blood mononuclear cells (PBMC) were obtained from patients and healthy donors (HD). PBMC were obtained after density gradient centrifugation (Ficoll-Lympholyte, Cederlane) as described before (53). This study was approved by Azienda Sanitaria Locale 3 (ASL, Genova, Italy) ethics board (N9-13, 2013) and after by Regione Liguria Ethics Board (Ethics Board id 4975, 2020). All patients gave written informed consent in according to the Declaration of Helsinki. PBMC of healthy donors were obtained from buffy coat (UO Centro Trasfusionale, IRCCS Ospedale Policlinico San Martino, Genova and IRCCS Bambino Gesù Children’s Hospital, Rome).

### Tissue Samples and Immunohistochemistry (IHC)

For each case of adenocarcinoma, all hematoxylin and eosin–stained slides were reviewed for confirmation of diagnosis; one block was then selected for adenocarcinoma tissue microarrays (TMAs) construction. For each block, five cores with a diameter of 1 mm were obtained from diverse areas of the tumor and randomly numbered from 1 to 5. From each TMA 5-μm sections were cut and stained with S100A9 (clone D5060, Cell Signaling Technology, Danvers, MA) on an automated staining platform (Benchmark Ultra, Ventana Medical Systems). An OptiView DAB IHC Detection Kit (Ventana Medical Systems). Stained sections were scanned with a Ventana iScan HT slide scanner (Ventana Medical Systems). The absolute numbers of S100A9-positive cells per mm^2^ were automatically counted in each core using QuPath version 0.2.0 (54).

### Antibodies and Flow Cytometry

For the evaluation of surface antigen expression the following monoclonal antibodies (mAbs) were used: CD3-APC-A700, CD19-APC-A700, CD56-ECD, PC7 and APC-A700, CD11b-FITC, CD33-PC7, HLA-DR-PE, CD14-ECD, CD45-KrO, CD66b-APC, CD15-APC (all Beckman Coulter), CD107a-eFLUOR660 (Invitrogen), CD275-, CD155-, CD85j-, Ceacam1-, CD39-APC (Miltenyi biotec). For intracellular evaluation the following mAbs were used: anti-IFN-γ-PE (BD, biosciences), anti-TNF-α-eFluor450 (Invitrogen). For MDSC immunostaining a custom Duraclone platform (Beckman Coulter) was used in order to standardize the protocol. After staining procedures cells were acquired at Cytoflex S and LX (Beckman Coulter) and analyzed with Cytexpert software (v2.2, Beckman Coulter), and FlowJo 10 (Starlab).

### Cell Isolation and Co-Culture Experiments and Cell Lines

PMN-MDSC cells were isolated by CD15 microbeads kit (Miltenyi Biotec) following manufacture instruction. NK cells were isolated as previously described (55), using NK isolation kit II (Miltenyi Biotec) or RosetteSep (StemCell technologies) (purity >95%). Freshly isolated NK cells either from patients or HD were immediately used or were cultured in 10% serum-supplemented RPMI 1640 medium (Lonza) supplemented with only IL-2 (100U/ml, Proleukin) for 48h referred as “short-term”. To obtain “activated” NK cells we performed long-term cultures (15-20 days) as previously described (56, 57).

Co-culture experiments were performed using effector cells (NK cells) cultured alone or in combination (1:1) with autologous or allogenic PMN-MDSC or in the presence of exosomes-derived PMN-MDSC (20ug). Co-culture experiments were performed in the absence or in the presence of α-TGF-β blocking mAb (R&D), α-arginase (N-hydroxy-nor-L-arginine, NOHA, 500mg/ml, Calbiochem, Germany), 1-Methyl-D-Tryptophane (1-MT indolamine-2,3-Dioxygenase inhibitor, 0,25mM Sigma Aldrich) and NS398 (N-[2-(Cyclohexyloxy)-4-nitrophenyl]methanesulfonamide, PGE2 inhibitor, 5µM Sigma Aldrich). After 48 hours PMN-MDSC were depleted from co-culture (as described before by CD15 microbeads kit) and the resulting NK cells were used to perform phenotypical and functional assays.

### Functional Assay

To assess the degranulation activity and cytokine production, NK cells were incubated with NALM-18 a Childhood B acute lymphoblastic leukemia cell line or A549 lung adenocarcinoma cells, as target cells at 1:1 effector:target (E:T) ratio for 4 hours in the presence of Monensin (2mM BD, GolgiStop) and Brefeldin A (1µg/ml BD, GolgiPlug) and CD107a mAb. To detect intra-cytoplasmic cytokines, after incubation, with target cells, NK cells were stained for surface markers, fixed and permeabilized with Fixation and Permeabilization Kit (BD Biosciences, New Jersey USA) and incubated with specific intracellular mAbs. To detect spontaneus degranulation a control sample without target cells was included.

Cell cytotoxicity assays was performed using as target cells NALM-18 cell line and as effector cells (short-term- or activated-NK cells) at different E:T cell ratios. In order to distinguish effector cells from target cells, NALM-18 cell line was stained with FITC cell tracker following manufacture instructions (Invitrogen). Iodure Propidio (PI) was added at the end of the co-culture (4 hours) in order to identify the percentage of target cell lysis. The calculation of specific lysis of NK cells was performed as described in (58).

### RNA Extraction and Gene Expression Analysis in NK Cells

Total RNA extraction from purified NK cells was performed with miRNeasy micro kit combined with on-column DNase I digestion following the manufacturer’s protocol (Qiagen GmbH, Hilden, Germany). For mRNA quantification, 300 ng of total RNA was reverse transcribed with random primers by using Super Script IV first-strand synthesis system following manufacturer’s instructions (Thermo Fisher Scientific, Wilmington, DE, U.S.A.). To explore a wide panel of genes, 150 ng of cDNA template per fill reservoir was loaded in 384-well TaqMan array microfluidic cards (Applied Biosystems, Foster City, CA, U.S.A) with a custom configuration focused on 92 human genes implicated in NK cell biology. Real time PCR analysis was performed with TaqMan Fast Advanced Master Mix (Applied Biosystems, Foster City, CA, U.S.A) on a QuantStudio 12k Flex instrument (Applied Biosystems, Foster City, CA, U.S.A). Expression values was calculated applying the relative threshold algorithm (Ct) with ΔΔCt method. Gene expression data were normalized by global normalization method using Relative Quantification app in Thermo Fisher Cloud (Thermo Fisher Scientific, Wilmington, DE, U.S.A.): the median Cq of all the assays in the PCR array card was calculated by software as the normalization factor, on a per sample basis.

### Exosome Isolation and Analysis

MDSC cells were plated at 4x10^6^ cells/ml in RPMI 1640 supplemented with 10% exosome-depleted Fetal Bovine Serum (FBS). After 48h, conditioned medium was collected and centrifuged at 300 x g for 5 min. Following centrifugation at 2000 x g for 15 min, supernatants were passed through a 0.22 µm filter and then exosome were pelleted by high-speed centrifugation (100’000 x g for 2 hours) (Optima X Optima XPN, Beckman, California, USA). Collected exosome were then washed with a large amount of Phosphate Buffer Saline (PBS) for an additional 1 hour and resuspended in PBS. Exosome samples were stored at -80°C until use. Exosome protein concentration was determined by Bradford Assay. For miRNA analysis exosomes were purified from culture supernatants by ultracentrifugation and RNA was extracted with miRNeasy micro kit (Qiagen). For each sample, the same amount of exosomal RNA (20 ng) was reverse transcribed with miRCURY LNA RT kit (Qiagen) and 3 µL of 1:30 diluted cDNA was amplified with miRCURY LNA miRNA Sybr Green system (Qiagen). For each sample, the synthetic spike-in UniSP6 was added as internal control to monitor cDNA synthesis and amplification efficiency. Outlier samples for UniSP6 expression were discarded from the analysis. All the real time PCR reactions were carried out in triplicate on a QuantStudio7 Flex instrument and data was analyzed with baseline threshold algorithm using QuantStudio Real time PCR software (Thermo Fisher Scientific).

### Expression Analysis on TCGA Dataset

RNA-sequencing expression data from 511 tumor samples of lung adenocarcinoma (luad) and 57 normal samples were retrieved from The Cancer Genome Atlas (TCGA) (59). The analysis was restricted to 57 individuals for which the complete sets of tumor and matched normal (normal tissue taken from the same patient) profiles were available, for a total of 114 samples.

We compared the NCR1 expression level on 57 luad samples and 57 matched-normal samples by applying a Student’s t-test for paired samples. Hierarchical clustering for KIR3DP1, FCGR3A, FCGR3B, NCR3, NCR1, KIR3DL2, CD15, S100A8, and S100A9 in luad samples and matched normal were obtained by clustering the expression profiles of genes according to rows and columns by using the Pearson correlation as distance metric and the complete-linkage as clustering method.

### Kaplan Meier Analysis on TCGA Dataset

To analyze the correlation between the expression level of genes and patient overall survival (OS), we exploited the RNA-sequencing data obtained from TCGA to split the entire cohort of lung adenocarcinoma patients (511 samples) into two groups (called low-expression and high-expression group) according to the upper and lower expression quartile. In particular, low- and high-expression groups refer to patients with expression levels of the given gene lower than 25th and greater than the 75th percentile of the expression levels distribution, respectively. For each patient cohort, the cumulative survival rates were computed for each gene according to the Kaplan-Meier (KM) method (60) on the clinical metadata provided by TCGA. For each gene, the survival outcomes of the two patients groups were compared by the log-rank test, showing a statistically significant p-value (< 0.05) if there exists a significant difference between the population survival curves.

### Statistical Analysis

Statistical analysis were performed with GraphPad Prism software (La Jolla, CA). In **Figures 1E** and **3A** we used nonparametric Mann–Whitney test; **Figure 2A** simple linear regression; **Supplementary Figure 1A** Student’s t-test for paired samples. **Figures 2B, E** and **3B**, **Supplementary Figures 1C, E** show the nonparametric Wilcoxon tests; **Figures 4E, F** one-way ANOVA plus post test for linear trend. A p value ≤ 0.05 was considered statistically significant. *p ≤ 0.05; **p ≤ 0.005; *** p ≤ 0.0005; **** p ≤ 0.0.00005. Where not indicated the data were not statistically significant.

## Data Availability Statement

The raw data supporting the conclusions of this article will be made available by the authors, without undue reservation.

## Ethics Statement

The studies involving human participants were reviewed and approved by Azienda Sanitaria Locale 3 (ASL, Genova, Italy) ethics board (N9-13, 2013) and after by Regione Liguria Ethics Board (Ethics Board id 4975, 2020). The patients/participants provided their written informed consent to participate in this study.

## Author Contributions

Designed experiments NT and PV. Performed the experiments NT, FB, SM ADP, AP, PFF, EM, and GB. Analyzed the data NT, PV, SM, ADP, and AP. Interpreted the results NT and PV. Wrote the manuscript NT, PV, SM, and LM. Provided samples from the patients FS and MGC. Followed patients enrolled in the study FS. Performed the molecular data set analysis PP and GF. Critically revised the manuscript LQ, PP and MCM. Provided intellectual input and revised the manuscript LM. All authors contributed to the article and approved the submitted version.

## Funding

This work was supported by grants from: Associazione Italiana Ricerca sul Cancro (AIRC) Investigator Grant ID 19920 (LM); Special Program Metastatic disease: the key unmet need in oncology 5 per mille 2018, ID 21147 (LM); 5 per mille Italian Ministry of Health (MCM), Ministero della Salute - Ricerca Corrente 2021 (PV). FB is recipient of fellowships awarded by AIRC. LQ was supported by European Union’s Horizon 2020 research and innovation program under the Marie Sklodowska-Curie Grant agreement no. 800924.

## Conflict of Interest

The authors declare that the research was conducted in the absence of any commercial or financial relationships that could be construed as a potential conflict of interest.

## Publisher’s Note

All claims expressed in this article are solely those of the authors and do not necessarily represent those of their affiliated organizations, or those of the publisher, the editors and the reviewers. Any product that may be evaluated in this article, or claim that may be made by its manufacturer, is not guaranteed or endorsed by the publisher.

## Supplementary Material

Supplementary Figure 1
**(A)** Boxplots of NCR1, FCGR3A, FCGR3B, NCR3, KIR3DL2, S100A8, S100A9, and CD15 expression level (logarithmic scale) in 57 lung adenocarcinoma samples (violet box) and 57 matched-normal samples (water blue box) retrieved from TCGA. P-value was obtained by applying a Student’s t-test for paired samples. **(B)** PBMC were analyzed *ex-vivo* by flow cytometry for the expression of specific markers that allow the identification of PMN-MDSC. Two representative experiments corresponding to 2 different patients with high and low percentage of PMN-MDSC. **(C, D)** NK cells were cultured alone (NK) or in the presence (ratio 1:1) of autologous or allogenic PMN-MDSC (cond.) derived from PB of lung tumor patients. After 48h of co-culture, PMN-MDSC were depleted from 1:1 co-cultures and the resulting NK cells used as effector cells in the different functional assays. **(C)** Percentages of killed mesothelioma or adenocarcinoma target cells isolated from lung tumor patients. Statistical analysis of 6 independent experiments. **(D)** Percentages of killed NALM-18 target cells by NK cells in autologous setting. One representative experiment out of 3 performed. **(E)** Cytokine production and degranulation capabilities of NK cells conditioned or not. Cells were analyzed after 4h of co-culture with NALM-18 target cells. Bars indicated percentage of median of cytokines production (IFN-γ and TNF-α) and degranulation (CD107a) of NK and cond. cells (n = 11). *p ≤ 0.05; **p ≤ 0.005; ***p ≤ 0.0005; ns, not significant.

Supplementary Figure 2
**(A, B)** Mean ± SEM of the indicated markers evaluated by flow cytometry on PBMC from **(A)** ex-vivo HD vs Lung tumor patients (lung) (n = 3) and **(B)** HD-NK cells cultured alone (NK) or after co-culture (48h) with PMN-MDSC (NK 1/1) of lung tumor patients (n = 4). **(C)** Expression of the indicated markers on PMN-MDSC of lung tumor patients by flow cytometry. **(D)** Fold of Inhibition of killing mediated by NK cells co-cultured with PMN-MDSC isolated from G-CSF mobilized HD (white bars, n = 4) and from PB of lung tumor patients (black bars, n = 10).
