# Supplementary figures and images for: Polymorphonuclear Myeloid-Derived Suppressor Cells Are Abundant in Peripheral Blood of Cancer Patients and Suppress Natural Killer Cell Anti-Tumor Activity
Source: Front Immunol. 2022 Jan 18;12:803014. doi: 10.3389/fimmu.2021.803014 (PMC8805733; doi:10.3389/fimmu.2021.803014)

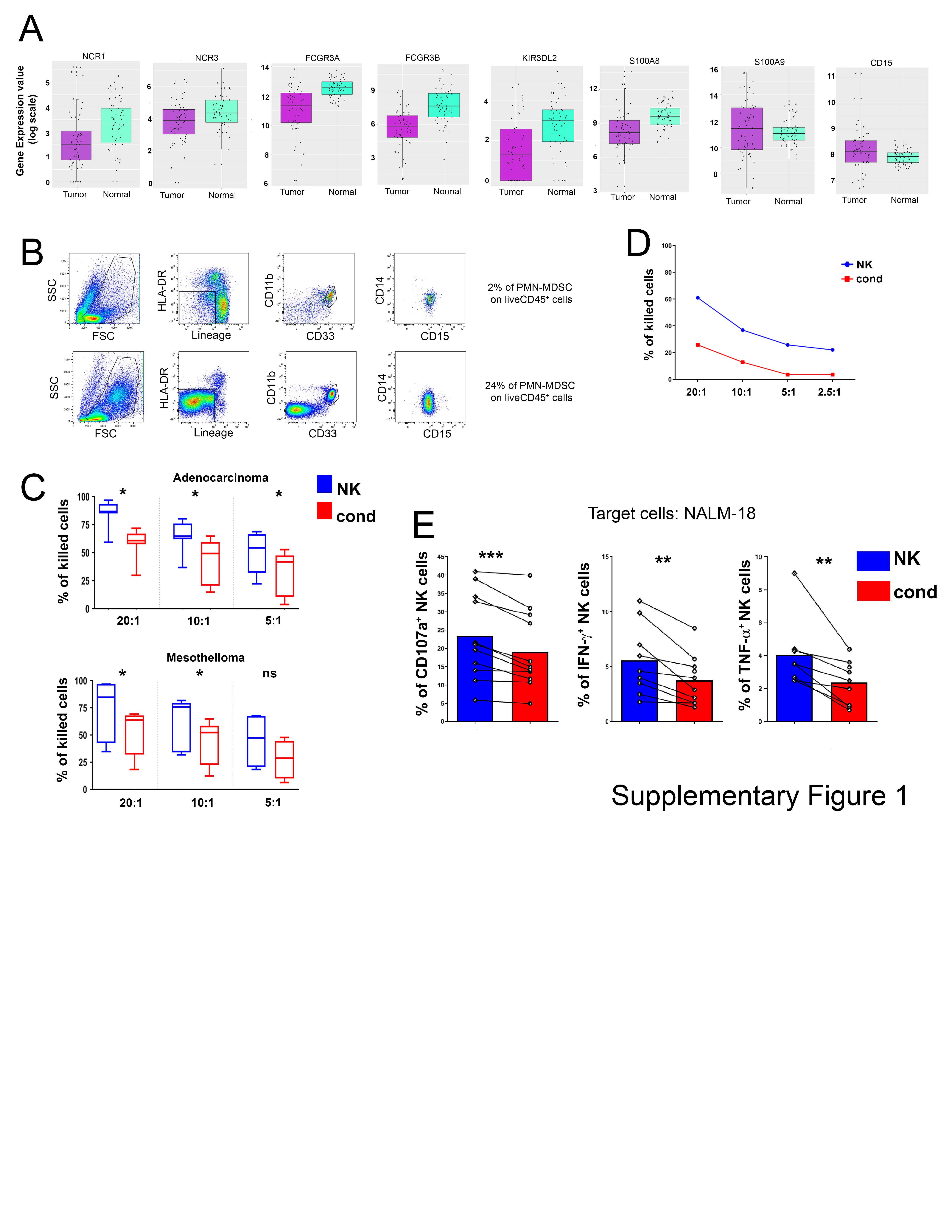

Supplement: Supplementary Figure 1 — (A) Boxplots of NCR1, FCGR3A, FCGR3B, NCR3, KIR3DL2, S100A8, S100A9, and CD15 expression level (logarithmic scale) in 57 lung adenocarcinoma samples (violet box) and 57 matched-normal samples (water blue box) retrieved from TCGA. P-value was obtained by applying a Student’s t-test for paired samples. (B) PBMC were analyzed ex-vivo by flow cytometry for the expression of specific markers that allow the identification of PMN-MDSC. Two representative experiments corresponding to 2 different patients with high and low percentage of PMN-MDSC. (C, D) NK cells were cultured alone (NK) or in the presence (ratio 1:1) of autologous or allogenic PMN-MDSC (cond.) derived from PB of lung tumor patients. After 48h of co-culture, PMN-MDSC were depleted from 1:1 co-cultures and the resulting NK cells used as effector cells in the different functional assays. (C) Percentages of killed mesothelioma or adenocarcinoma target cells isolated from lung tumor patients. Statistical analysis of 6 independent experiments. (D) Percentages of killed NALM-18 target cells by NK cells in autologous setting. One representative experiment out of 3 performed. (E) Cytokine production and degranulation capabilities of NK cells conditioned or not. Cells were analyzed after 4h of co-culture with NALM-18 target cells. Bars indicated percentage of median of cytokines production (IFN-γ and TNF-α) and degranulation (CD107a) of NK and cond. cells (n = 11). *p ≤ 0.05; **p ≤ 0.005; ***p ≤ 0.0005; ns, not significant. [file Image_1.jpeg]

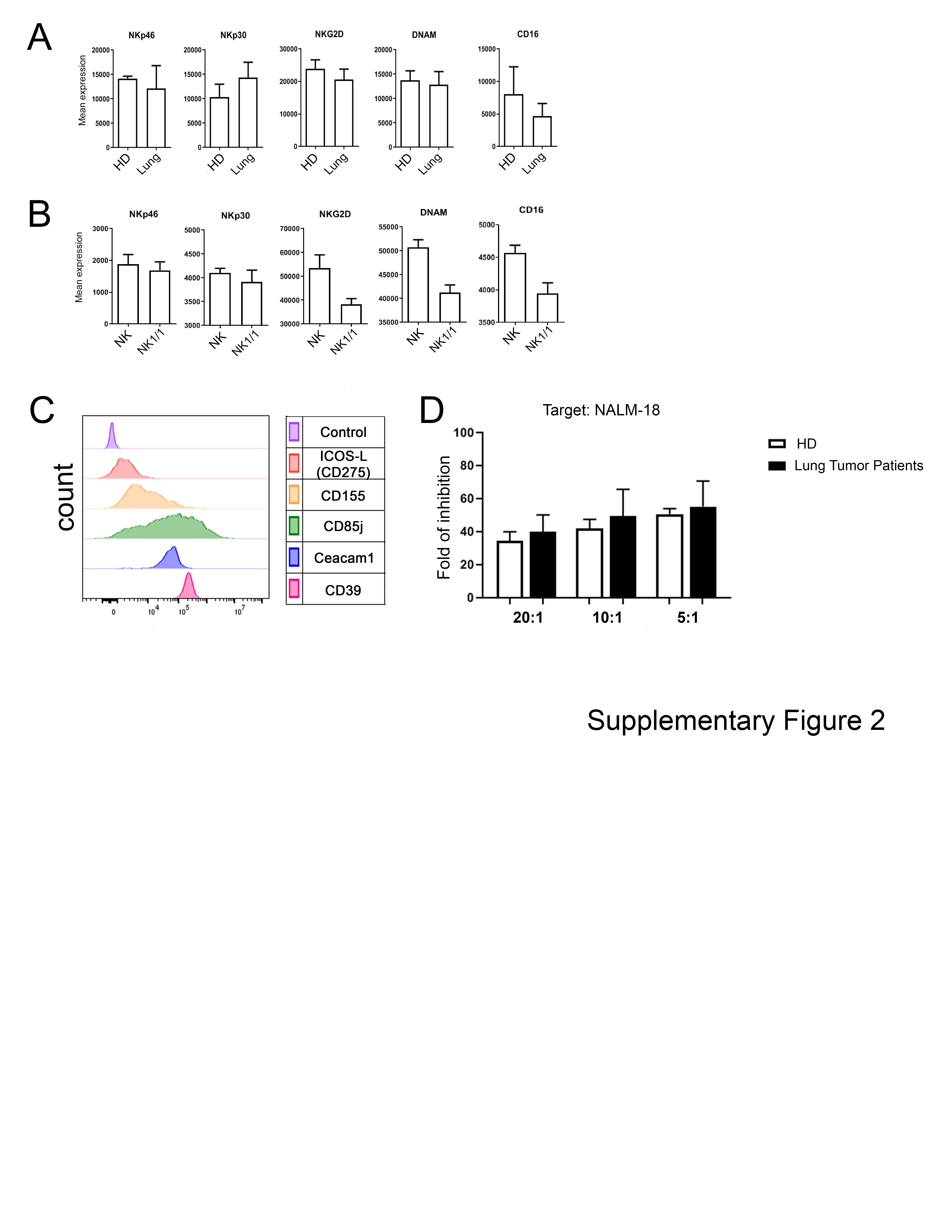

Supplement: Supplementary Figure 2 — (A, B) Mean ± SEM of the indicated markers evaluated by flow cytometry on PBMC from (A) ex-vivo HD vs Lung tumor patients (lung) (n = 3) and (B) HD-NK cells cultured alone (NK) or after co-culture (48h) with PMN-MDSC (NK 1/1) of lung tumor patients (n = 4). (C) Expression of the indicated markers on PMN-MDSC of lung tumor patients by flow cytometry. (D) Fold of Inhibition of killing mediated by NK cells co-cultured with PMN-MDSC isolated from G-CSF mobilized HD (white bars, n = 4) and from PB of lung tumor patients (black bars, n = 10). [file Image_2.jpeg]
